# Catheter‐related thrombosis diagnosed by computed tomography

**DOI:** 10.1002/ccr3.801

**Published:** 2017-02-03

**Authors:** Yohei Yamauchi, Akira Baba, Yumi Okuyama

**Affiliations:** ^1^ Department of Internal MedicineTokyo Dental College Ichikawa General HospitalChibaJapan; ^2^ Department of RadiologyTokyo Dental College Ichikawa General HospitalChibaJapan

**Keywords:** Catheter‐related thrombosis, computed tomography

## Abstract

Catheter‐related bloodstream infection can be detected with CT. Although it is not mandatory for its diagnosis, it is strong evidence as a cause of fever if detected incidentally.

A 76‐year‐old man suspected of amyotrophic lateral sclerosis was referred to our hospital, complaining of eyelid dropping and limb muscle weakness. Due to hypoxemia, he was ventilated first and then tracheostomy was performed. Central venous catheter (CVC) was placed via the right femoral vein for intravenous hyperalimentation because of dysphagia due to muscle weakness. His dyspnea improved with medications, but high fever developed. Contrast‐enhanced computed tomography (CECT) revealed thrombosis containing gas bubbles around the tip of CVC without other findings of infection (Figure 1). The catheter was removed and antibiotic therapy was applied, and the fever subsided immediately. The same bacteria were isolated from blood cultures and catheter tip culture. Infected thrombosis can be identified by CECT as the filling defect with air density in tips of CVC 1; thus, CECT is powerful for correct diagnosis of catheter‐related infection. In general, for detecting the cause of unidentified fever, whole‐body CECT is often performed. In this case, catheter‐related thrombosis was detected by radiologist, without clinicians noticing it. Clinician, not radiologist, who is not familiar with imaging diagnosis, should be careful to check the artificial parts, especially CVC tip, in addition to normal anatomical parts in investigation of patients with fever of unknown origin.

**Figure 1:**
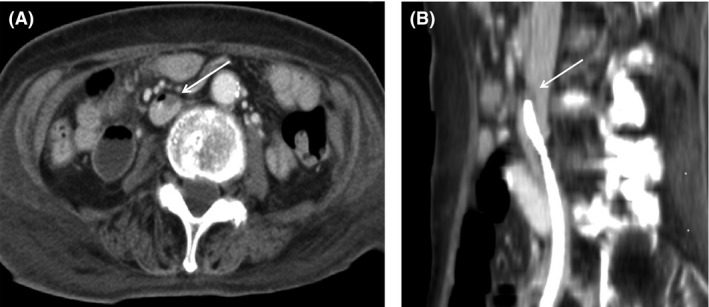
(A) Axial CECT. (B) Curved MPR along IVC. CECT and curved MPR along IVC revealed thrombosis containing gas bubbles around the tip of CVC (arrow) without other findings of infection.

## Authorship

YY: drafted the article. YY, AB, and YO: participated in critical review and revision of the articles. YY, AB, and YO: approved the article. YY, AB, and YO: are accountabile for all aspects of the work.

## Conflict of Interest

None declared.
